# A Perspective Analysis of Dams and Water Quality: The Bui Power Project on the Black Volta, Ghana

**DOI:** 10.1155/2018/6471525

**Published:** 2018-10-01

**Authors:** Samuel Fosu Gyasi, Bismark Boamah, Esi Awuah, Kenneth Bentum Otabil

**Affiliations:** ^1^Department of Basic and Applied Biology, School of Science, University of Energy and Natural Resources, Sunyani, Ghana; ^2^Department of Energy and Environmental Engineering, School of Engineering, University of Energy and Natural Resources, Sunyani, Ghana; ^3^Department of Civil Engineering, School of Engineering, Kwame Nkrumah University of Science and Technology, Kumasi, Ghana

## Abstract

Large dams play an important role in promoting economic and social development in many countries. However, the construction of such dams can have a detrimental effect on the environment. The aim of this study was to investigate perceptions of drinking water quality among inhabitants of selected communities within the Bui Dam environs. With the help of questionnaires, 100 respondents from communities “near to the dam” were randomly selected and interviewed. Their responses were compared with another 100 respondents selected from “far from the dam” communities. These were augmented with in-depth interviews, focus group discussion, and personal observation. Analysis of the results showed that, there were greater proportions (31%) of the participants who lived in “near communities” within the age category 20–25 compared to 19% of their “far communities” counterparts. There were significantly greater proportions of female respondents in the “near to the dam” (57%) compared to respondents in the “far from the dam” communities (52%). The study further showed that the perception of risk of consuming contaminated drinking water was more common among “far from the dam” communities (odds ratio = 4.57). The perception of the quality of water based on some physical properties was investigated as part of our study. Analysis of the results showed that significantly greater proportion of the “far from the dam” communities (35%) perceived their water had an objectionable smell compared to 7% of inhabitants of their other counterparts (*p* value = 0.001). The study further showed that significantly greater proportion of the study participants in the far from the communities perceived that their water had colour (65%) and they did not drink water from any other source (63%) apart from their stream. The study demonstrated that generally, inhabitants within the study communities perceived the construction of the Bui Power Project has negatively affected their drinking water quality.

## 1. Introduction

Development of rivers for large dams have emerged as one of the most significant and visible tools for the management of water resources [1]. Large dams play an important role as they promote economic and social development. They also provide important services such as electricity generation, water supplies, and flood control [2]. However, proposals for new dams in many countries have aroused intense opposition [3] with many social and economic arguments used against its construction. A major reason for such arguments is the fact that large dams produce major ecological changes in river ecosystems [3]. Dams have impacts on both upstream and downstream ecosystems [4]. They constitute obstacles for longitudinal exchange along rivers and disrupt many natural environmental processes. Flooding upstream of dams results in the permanent destruction of terrestrial ecosystems through inundation. All terrestrial plants and animals disappear from the submerged area [4].

For some time now, flooding of dam has created a plethora of disturbances in the ecosystem, varying from enormous ecological and productivity related changes [5]. This massive land modification occurs according to the physical and biological characteristics of the site and the management regime of the dam. Floods have the tendency to cause hydraulic disturbances that determine the composition of biotic communities within the channel, the riparian zone and the flood plain [6]. Water storage in reservoirs induces physical, chemical, and biological changes in stored water. Elsewhere during arid climates, water below dam causes salinization arising from increased evaporation and particularly problematic in areas of marine sediments.

Since 1965, the government of Ghana started the construction of Akosombo Dam, forming Lake Volta, the largest water storage reservoir in Africa and even the world [7]. This dam provided the energy needs of a then growing country with relatively fewer people and less industries. In more recent years, due to rapid population growth, industrialization, and urbanization, a serious energy deficit has emerged. This resulted in an urgent need to build another hydroelectric power dam, the Bui Hydroelectric Dam to reduce the deficit. Upon completion, the Bui Hydroelectric Dam generates electricity and, in addition, supplies water for domestic and industrial uses. In addition, water is harvested from this dam to irrigate agricultural lands to boost food production. However, the benefits of this project cannot be without considerable social, economic, and environmental costs, as has been the case with dams elsewhere in the world and even with the Kpong and Akosombo Dams in Ghana. Meanwhile, following the completion and operation of the Bui Dam, there has been numerous reports by some sections of the media and some major stakeholders that some inhabitants living close to the Bui Hydroelectric Project area perceive that the quality of their drinking water has been compromised. Till date, there are no empirical data in published articles to ascertain the veracity of this assertion by inhabitants of the Bui dam environs although different research conducted by other investigators in other regions supports such assertion [8, 9]. There is therefore an urgent need for a study such as this to investigate the perception of inhabitants on drinking water quality of some surface water after the construction of the Bui Dam. The main aim of this study therefore was to investigate the perception of the quality of drinking water among inhabitants of some selected communities within the Bui Dam environs. These perceptions are important as they can influence the relationship between the dam operators and the host community. This study will also provide the basis for studies to investigate those perceptions.

## 2. Materials and Methods

### 2.1. Study Area and Population

The study was carried out within some selected communities upstream and downstream of the Bui Dam environ in the Bole district (northern region) and the Banda-Ahenkro district (Brong-Ahafo Region) in north-western Ghana, approximately 150 km upstream of Lake Volta. The study area lies between latitudes 8˚09′-8°.16′ North and 2˚01′-2°. 15′ longitude West. The vegetation in the Bui area consists of about 60% savannah woodland, 10% riparian forest, and 30% grassland. There are about 45 communities surrounding the Bui Hydropower project with an estimated population of about 29,287. The most dominant occupation of the inhabitants is fishing [10].

### 2.2. Selection of Study Communities

**Figure 1:**
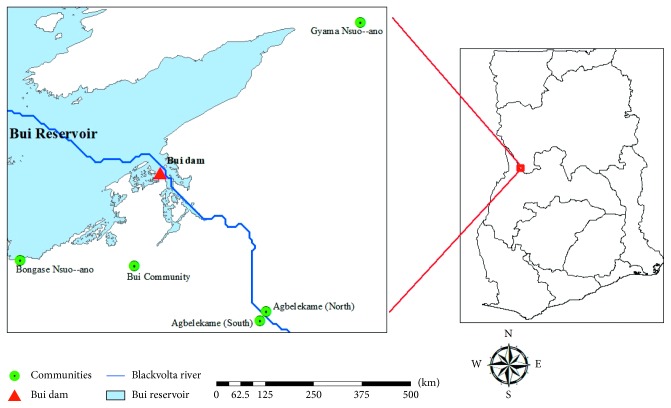
Showing the GPS location of study communities where samples were taken.

Communities for the study were systematically selected based on their farness or closeness to the dam. These included Agblekame South, Bongase Nsuo-Ano (Banda-Ahenkro), and Agblekame North, Gyama Nsuo-Ano (Bole Bamboi), as shown in Figure 1 below.

### 2.3. Study Design and Communities Selection

The study adopted a longitudinal study approach with responses from respondents domiciled in close communities compared with those of their far community counterparts. On the basis of this, Agblekame South and Agblekame North were designated to be the “far communities” while Gyama Nsuo-Ano and Bongase Nsuo-Ano were designated as “near communities.”

### 2.4. Study Methodology

The study used a combination of desk studies, checklists, and interviews to collect primary data. With the help of house to house visits, respondents were randomly selected and interviewed. Structured questionnaires designed for respondents included both open and closed-ended. A total of 200 questionnaires were administered to respondents from the 4 communities with an approximate population size of 2,400 inhabitants during the entire study. Hundred (100) questionnaires were administered randomly to interview respondents selected from the near communities whose responses were compared with another 100 selected from their far communities counterpart. The questions asked were based on sociodemographics, household water usage patterns, and households' hygienic behaviour as perceived by respondents (Lagardere, 2007). These were augmented with in-depth interviews, focus group discussions (FGDs), and personal observation. The use of multiple complimentary methods made it possible to triangulate and eliminate bias that could occur if only one method was employed (Adubofour et al., 2012). The study was conducted between January and June 2015. Respondents were interviewed in Twi (the predominant local language of the inhabitants), and these were later transcribed into English language.

## 3. Data Analysis

Data from answered questionnaires were manually entered in Microsoft Excel (2013). With the help of GraphPad Prism 5 software, categorical variables were analysed using chi-square at 95% confidence interval with *p* value ≤ 0.05 considered significant. Using Linear regression odds ratio (OR), the “perceptions” in terms of the measure of association between a possible exposure and a perceived risk were estimated.

## 4. Results

In this study, the social demography of respondents was assessed based on ones' location with respect to the Bui Dam. Analysis of the results showed that there were greater proportions (31%) of the participants who lived in “near communities” that fell within the age category 20–25 compared to 19% of their “far communities” counterparts (Table 1). A greater proportion of the “close to the dam” communities respondents (12%) were adults (aged over 50) compared to their counterpart from the far from the dam communities although the difference was not significant. In terms of gender, there were significantly greater number of female respondents in the “near to the dam” (57%) compared to respondents in the “far from the dam” communities (52%) as shown in Table 1 below. Analysis of the marital status of the respondents in the study showed greater proportions (67%) of respondents from Bole (near to the dam) being married compared to only 43% of their Banda-Ahenkro (far from the dam) counterparts (*p* value = 0.0006) as shown in Table 1 below.

Analysis of the level of education of respondents based on whether a respondents was living near or far from the Bui dam was assessed. Results of the analysis showed that, fewer proportions of respondents in the Banda-Ahenkro (far from the dam) (25%) had some form of basic education (primary education) compared to their Bole district counterpart (near to the dam) (47%) and this was significant (*p*=0.012) with an odds ratio of 3.8 (Table 1). The study investigated the occupation of respondents based on one's location with respect to the dam and analysis of the results showed that, significantly greater portion of respondents from “near to the dam” communities (65%) were fishermen compared to 49% of “far from the dam” downstream community respondents (49%) (Table 1) (*p*=0.022) as shown in Table 1 below.

The study further showed that the perception of risk of consuming contaminated drinking water was more common among “far from the dam” communities due to their location from the dam (odds ratio = 4.57) compared to those of the “close to the dam” communities (Table 1).

The study further investigated the general perception of drinking water quality of respondents in selected communities in the 2 districts, i.e., Banda-Ahenkro (far from the dam) and Bole district in the Brong Ahafo (near to the dam) and Northern regions, respectively. To achieve this, the study investigated the source of water for drinking and for domestic purposes of respondents from both near and far communities. Analysis of the responses showed that a greater proportion (87%) of the “far from the dam” communities relied on water from streams in their communities compared to of their “near to the dam” counterparts (67%) as shown in Table 2 below, and this was significant (*p* value = 0.0008). With respect to the reason for their choice of drinking water sources, 39% of “far from the dam” inhabitants' perceived proximity as the deciding factor compared to only 23% of inhabitants from the “near to the dam” communities (Table 2).

When respondents in the study were asked whether or not their source of drinking water was treated, the results showed that respondents from the “far from the dam” communities were perceived to be about 5 times at risk (Odds ratio 4.8) due to failure to treat drinking water (Table 2). The question of as to whether or not respondent saw the need to treat their water before drinking was also posed to study participants. Analysis of the results revealed that a relatively fewer proportion (43%) of the “far from the dam” respondents were of the opinion that treatment of water before use was needless as against 45% of their “near to the dam” counterparts. Water storage behaviour of respondents in the 2 regions were compared, and the results showed that greater proportion of “far from the dam respondents” (Brong Ahafo Region) inhabitants (97%) stored their water compared to their “near to the dam” (northern region) counterparts (86%). The far from the dam communities also perceived the presence of debris in stored water, and the difference was significant (Table 2).

The perception of the quality of water based on some physical properties was investigated as part of our study. Analysis of the results showed that, in terms of the smell of the water, significantly greater proportion of the “far from the dam” communities (35%) perceived that their water had an objectionable smell compared to 7% of inhabitants, their other counterparts (*p* value = 0.001). The study further showed that significantly greater proportion of the study participants in the far from the communities perceived their water had colour (65%) and they did not drink water from any other source (63%) apart from their stream (Table 2).

The water usage patterns, water related tropical diseases infections, and the behavioural pattern of inhabitants living in 2 districts around the Black Volta were investigated. Analysis of the water usage patterns of the respondents showed that greater proportions of inhabitants from the “far from the dam” communities (18%) had daily contact with their stream compared with just 5% of the individuals from the “near to the dam,” and this was significant (*p*=0.004). Majority of the “far from the dam” communities (97%) also perceived that the siting of the dam had a negative effect on their health compared to those living in the “near to the dam” (86%) (Table 3 below). The study also showed that greater proportions of the “near to the dam” dwellers (23%) reported having been clinically diagnosed of Schistosomiasis compared to their counterparts (2%) as shown in Table 3. With respect to malaria, the study showed that respondents from the far communities perceived that they were 4 times more at risk of getting malaria compared to those in the near end of the dam (Table 3). Our investigations further demonstrated that with regards to common symptoms of diseases frequently experienced, 11% of the “near to the dam” inhabitants often experienced coughing, whilst 37% had some form of diarrhea. Meanwhile, 67% of the inhabitants often passed urine with blood and impaired vision was common in 3% of the people. These proportions were generally higher in the “near to the dam” communities compared to their “far from the dam” counterpart communities (Table 3).

Although inhabitants from both locations engaged in habitual hand washing at least 2 times a day, greater proportion of “near to the dam” dwellers (93%) washed their hands before eating with bare hands compared to their “far from the dam” counterparts (65%), and this was significant with (*p*=0.022; OR = 0.52). Analysis of the responses on “*how many times one visited the bathroom*' showed that there were no significant differences in this particular behaviour based on ones' location (Table 3). More so, our results demonstrated that fewer proportions of “far from the dam” respondents (29%) patronized the community dump sites compared to 71% of their “near to the dam” communities, and the difference was statistically significant (*p* < 0.0001; OR = 0.0217) as seen in Table 3. The study also showed that inhabitants of “far from the dam” communities were 5 times more likely to dump their refuse in open spaces around their immediate surrounding compared to inhabitants from “near to the dam” communities as shown in Table 3 (*p*=0.0004; OR = 4.94).

The study also analysed the feacal discharge management practices of the participants. It was evident from the results that majority “near to the dam” respondents (66%) defecated openly compared to only 27% of their counterpart as shown in Table 3 below (*p* < 0.0001; OR = 5.24).

## 5. Discussion

Our study showed a high number of females in the “near to the dam” communities than “far from the dam” counterparts, although there was no significant difference in their perception of the quality of drinking water. The high number of females in “near to the dam” could be due to the emergence of new female-friendly business opportunities in the area. Some studies have found that women tend to perceive a higher risk than men, particularly in cases of technological health and safety implications of risks [11, 12]. However, our study could not find any difference in the perception of risk among the gender. The results of our study is however similar to studies by other investigators which demonstrated that women and men do not show significant difference when it comes to local environmental issues. For example, El-Zein et al. found that gender differences in expressing views about environmental concerns were a matter of sociocultural division of labor, while Howel et al. found little or no differences between gender concerning their views on the links between air pollution and health [13, 14]. More so, Grasmuck and Scholz in a study elsewhere found no gender effect when examining the perception of heavy metal soil contamination in a community in Northwest Switzerland [15].

In this study, respondents from “near to the dam” communities who had formal education had a higher degree of knowledge of understanding on the potential impact of dams on their drinking water. They were also well abreast with activities in their environment that could result in damaging their drinking water quality. It is generally known that people who are more educated tend to be more water quality conscious. The findings of this study are however different from those of a study conducted by Larson and Edsall, where the level of education did not commensurate with knowledge of water quality concerns [16]. However, it can be argued that, in their study, most respondents were between the ages 40 to 50 and had received their education in 1970's, where water issues in the country were generally not considered as a problem nor included in educational programs. Thus, they could not recount any educational experience related to water. Interestingly, some people in the study by Larson and Edsall still referred to water as an inexhaustible resource based on their previous education and experience. In these respondents, the old ideology about water seemed to be entrenched and difficult to change [16].

The present study also showed that the main occupational activity of both “near to the dam” and “far from the dam” communities was fishing. Some perception studies, especially those focusing on the difference in views among rural and urban residents about particular environmental aspects, take into consideration the role of place-dominant activities. For example, Salka found that as the percentage of workers in the natural resource industry increased, environmental initiatives faced more opposition [17]. Similar results are mentioned by Houghton et al. who found that persons working in extractive sector show lower levels of environmental concerns than those working in agriculture [18]. The preference of rural people for community economic growth over the environmental degradation is a function of their dependence on the extraction and use of natural resources [19].

Our study further showed that the “far from the dam” communities relied heavily on streams as sources of drinking water as opposed to their “near to the dam” communities who had other sources such as sachet and bottled water. This phenomenon could be because the “near to the dam” communities were generally more enlightened than their “far from the dam” counterpart, thus increasing their access to better drinking water. The sustainable development goal 6 seeks to ensure universal access to safe and affordable drinking water for all by 2030 [19], though several challenges are anticipated. Despite the steadily increasing supply of drinking water throughout the world, water quality continues to be of concern in many developing countries and, to a lesser extent, in developed nations [20]. In developing countries, many urban areas face the unevenness between supply and demand of reliable supply of good quality drinking water [21]. Improved access to water supply and sanitation remains one of the primary ways of addressing poor health in developing countries. Since 1990, access to drinking water coverage has expanded in sub-Saharan Africa by about 22%, though it still remains low, with only 60% of the population served [19]. The challenge for water improvements also remains greater for most sub-Saharan African countries, where coverage is mostly below average. In many developing countries, insufficient access to clean water and adequate sanitation and the resulting health issues are acute problems. Every year, the lack of safe water, sanitation, and hygiene cause about 88 % of deaths from diarrheal diseases, accounting for 1.5 million such deaths—majority of which occur among children under the age of 5 [22]. To win any health battles, in developing countries, therefore, secure, clean water and sanitation facilities for all should be a government priority. Health psychologists recognize the perceived risk of illness as one of the most important factors in a household's precautionary behaviours [23].

This study again showed that majority of “far from the dam” respondents did not treat their drinking water compared to their upstream counterpart. Without safe public water supplies, households' health and well-being are at risk. Domestic water treatment has been shown to be one of the most effective means of reducing the risks and costs associated with preventing water-borne diseases, especially diarrhea [24]. However, despite the importance of increasing water quality through domestic treatment, empirical research remains scarce on the relationship between water treatment and factors such as risk perception that drive this decision. There appear to be few studies focusing on the above issues. Notable exceptions are those by Cai et al., Jakus et al., and Nauges and van den Berg [25–27]. Nauges and van den Berg studied the perception of health risk and averting behaviour for nonpipe water sources in Sri Lanka [27]. Jakus et al. examined the rationale behind people in the United States (US) buying bottled water [26], while Cai et al. explored altruistic averting behaviour of removing arsenic risk in drinking water in the US [25].

This result confirmed the important role perceived risk plays in changing health behaviour, as found in earlier studies that provided risk information [28, 29]. These results also resonates with previous findings by Nauges and van den Berg that households were aware that treating nonpiped water lowers the risks related to the consumption of unimproved water [27]. The results of their study further suggested that the probability of treating water decreases if the head of the household or the respondent was male. Males were 21% less likely than females to treat nonpiped unimproved drinking water. One possible explanation was that women, who were generally responsible for taking care of children in the study areas, might have found it more worthwhile to treat water to avoid water-borne diseases, for example. These results are in line with experimental measures of risk aversion studies, where it is often found that women are more risk-averse than men [30].

Our findings on respondent perception of the quality of water with respect to the construction of the Bui dam based on some physical properties showed that significantly greater proportion of the “far from the dam” communities perceived their drinking water had an objectionable smell compared to “near to the dam” communities counterpart. Inhabitants perceived that the creation of the Bui Dam had caused great loss to vegetation and caused aquatic and terrestrial organisms to be submerged. However, the deterioration of the water quality in the downstream of the Bui subbasin could be attributed to the decay of dead animals and plants in the water from the time the research was being carried out. The physicochemical parameters of some dams and reservoirs have been studied in pre- and postimpoundment conditions [31]. However, studies on the tropical regulated downstream river of dams are limited though it is also subjected to major environmental impacts ranging from downstream morphological changes to change in biodiversity of the ecosystem. Downstream impacts of the dam can sometimes extend up to a distance of about 100 km from the dam site [32], although the intensity of the impacts tends to decline with increasing distance from the dam site. Dams can also change downstream hydrology by altering the flow pattern which and this changes the water quality of the downstream river [33].

The perceived high prevalence of water-related diseases in younger children and older females could be due to their regular visit to their source of water, fetching with buckets for drinking and/or other domestic purposes (older women) or to swim for younger children. In the Black Volta basin of Bui Dam, inhabitants perceived that impoundment of Bui River created the opportunity for growth of aquatic weeds in some areas and also the influx of immigrants (fishermen already infested with *Bilhazia* disease) schistosomiasis (also known as bilharzia, disease caused by parasitic worms of the genus *Schistosoma*) [34]. The economic and health effects of schistosomiasis are considerable, and the disease disables more than it kills. In children, schistosomiasis can cause anaemia, stunting, and a reduced ability to learn, although the effects are usually reversible with treatment. Chronic schistosomiasis may affect people's ability to work and in some cases can result in death. The number of deaths due to schistosomiasis currently is difficult to estimate because of hidden pathologies such as liver and kidney failure as well as bladder cancer. Elsewhere, the emergences or reemergence of schistosomiasis has resulted from large-scale hydropower projects, e.g., Gezira-Managil Dam (Sudan), Aswan Dam (Egypt), Melkasadi Dam (Ethiopia), and the Danling and Huangshi Dams (China) [35]. Similarly, changes in water level and downstream sediment deposition resulting from the building of the Three Gorges Dam in China seemed to increase the schistosomiasis transmission season within the marshlands along the middle and lower reaches of China's Yangtze River [36].

From our study, “far from the dam” dwellers reported relatively low biting frequency of the black fly insects after the construction of the Bui Dam. This is positive because Black Volta basin of the Bui Dam is known to be heavily infested with black fly insects. The reportedly reduced abundance of the black fly population could be attributed to the Bui river impoundment in the Bui subbasin of the Black Volta. These black flies breed along fast-flowing rivers and streams, close to remote villages located near fertile land where people rely on agriculture [37]. The black flies are vectors of river blindness, a neglected tropical disease (NTD) caused by infection with the parasitic worm *Onchocerca volvulus*. The burden of the disease has been reduced by prevention efforts, including control of the fly vector and periodic ivermectin therapy in at-risk individuals.

This study also proved that “far from the dam” inhabitants' perceived they were 4 times more at risk of getting sick with malaria than their “close to the dam” counterparts. This could be attributed to the fact that the people living in the “near to the dam” communities resided closer to the Bui Dam where there was a high possibility that inhabitants could have contact with mosquitoes. Malaria is a mosquito-borne infectious disease affecting humans and other animals caused by parasitic protozoans (a group of single-celled microorganisms) belonging to the *Plasmodium* type [37, 38].

Further, our study also showed that the practice of environmental hygiene was better in “far from the dam” communities as against their “near to the dam counterpart” counterparts. Though “near to the dam counterpart” inhabitants had containers they could dispose their solid waste into, they often disregarded this activity by disposing their solid waste directly in their environs with key examples being fishmongers. The worst offenders of these were fishermen who lived closer to the river banks and depended on this source of water for their livelihood. Hygiene has been found to be a primary preventive measure against diarrhea [38]. Even though water supply and sanitation impact on diarrhea, hygiene measures further minimize the effect of poor water supply and poor sanitation on diarrhea [22]. A lot of studies have pinpointed the effects of hygiene on disease transmission. Mara (2003) suggested that hygiene is potentially one of the most effective means of reducing the global burden of diarrhea diseases in children [39]. Waste that is not properly managed, especially excreta and other liquid as well as solid waste from households and communities, constitutes a serious house health hazard which tends to spread infectious diseases. Unattended waste lying around attracts flies, rats, and other creatures that in turn spread disease. Normally, this is a result of the wet waste that decomposes and releases bad odour [39]. The category of people who are more likely to dispose solid waste include the population in areas where there is no proper waste disposal sites, especially school children and workers in facilities producing toxic and infectious materials. Organic domestic wastes also pose a serious threat since they ferment creating conditions favourable to the survival and growth of microorganisms.

## 6. Conclusion

This study sought to investigate the perceptions of the participants about the impacts of the construction of the Bui Dam on their communities. The study demonstrated that generally, inhabitants “near to the dam” communities perceived the construction of the Bui Power Project has negatively affected their drinking water quality as well as their health. The perception of the negative impacts of the dam increased with proximity to the dam site. However, since perceptions of risks do not translate into empirical evidence on the actual impact of the dam, it is important to urgently carry out studies needed to ascertain whether these perceptions are the reality so that remediating measures could be put in place.

## Figures and Tables

**Table 1:** Respondent demographic data stratified by location in the Banda and Bole Districts of Ghana.

Variables	% total (200)	% far from the dam communities (100)	% near to the dam communities (100)	*p* value	Odds ratio (OR)
*Age*					
Less than 20	10 (5.0)	3 (3.0)	7 (7.0)	0.194	0.41
20–25	50 (15.0)	19 (19.0)	31 (31.0)	0.05	0.522
26–30	52 (16.0)	20 (20.0)	32 (32.0)	0.053	0.37
36–40	39 (19.5)	15 (15.0)	24 (24.0)	0.108	0.558
46–50	17 (8.5)	8 (8.0)	9 (9.0)	0.7998	0.879
Over 50	32 (16.0)	12 (12.0)	20 (2.0)	0.1228	0.546
*Sex*					
Male	80 (40.0)	43 (43.0)	48 (48.0)	0.04777	0.8173
Female	120 (60.0)	52 (52.0)	57 (57.0)	0.4777	1.224
*Marital status*					
Married	110 (55.0)	43 (43.0)	67 (67.0)	0.0006	0.37
Single	56 (28.0)	19 (19.0)	37 (37.0)	0.0046	0.399
Divorce	20 (10.0)	9 (9.0)	11 (11.0)	0.637	0.8
Separated	4 (2.0)	1 (1.0)	3 (3.0)	0.312	0.33
Widowed	10 (5.0)	5 (5.0)	5 (5.0)	1	1
*Educational level*					
Primary	72 (36.0)	25 (25.0)	47 (47)	0.0012	0.38
JHS	52 (26.0)	21 (21.0)	31 (31.0)	0.106	0.59
SHS	16 (8.0)	5 (5.0)	11 (11.0)	0.118	0.43
Tertiary	6 (3.0)	2 (2.0)	4 (4.0)	0.41	0.49
Never	56 (28.0)	23 (23.0)	33 (33.0)	0.115	0.6
*Occupation*					
Farmer	20 (10.0)	16 (16.0)	4 (4.0)	0.005	4.57
Fisherman	114 (57.0)	49 (49.0)	65 (65.0)	0.022	0.52
Apprentice	4 (2.0)	2 (2.0)	2 (2.0)	1	1
Trading	36 (18.0)	11 (11.0)	25 (25.0)	0.01	0.37
Government worker	0 (0)	0 (0)	0 (0)	0	0
Galamsey operator	10 (5.0)	8 (8)	2 (2.0)	0.05	4.3
Unemployed	16 (8.0)	12 (12.0)	4 (4.0)	0.04	3.27

OR refers to odds ratio; *p* value refers to level of significance. The “far from the dam” communities were Agblekame North and Agblekame South, while “near to the dam” communities were Gyama Nsuo-ano and Bongase nsuo-ano.

**Table 2:** Respondents' general knowledge on their drinking water quality based on location.

Variables	% total (200)	% (100) far from the dam communities	% (100) near to the dam communities	*p* value	Odd ratio
*What is the source of drinking water?*					
Stand pipe	0 (0)	0 (0)	0 (0)	0 (0)	0 (0)
Borehole	10 (5.0)	6 (6.0)	4 (4.0)	0.52	1.53
Stream	144 (72)	87 (87.0)	67 (67.0)	0.0008	0.3
*Why this source?*					
Source is closest	62 (31)	39 (39)	23 (23)	0.0144	0.47
Water is reliable	0 (0)	0 (0)	0 (0)	0 (0)	0 (0)
Water is clean	0 (0)	0 (0)	0 (0)	0 (0)	0 (0)
Other	138 (69)	49 (49.0)	89 (89.0)	<0.0001	0.119
*Is your drinking water treated?*					
Yes	23 (11.5)	5 (5.0)	18 (18.0)	0.004	0.24
No	175 (87.5)	95 (95.0)	80 (80.0)	0.0013	4.8
*Why would you/would not treat drinking water?*					
Source is polluted	10 (5.0)	3 (3.0)	7 (7.0)	0.194	0.41
Source is clean	50 (15.0)	19 (19.0)	31 (31.0)	0.05	0.522
Less likely to get sick	52 (16.0)	20 (10)	32 (11.0)	0.053	0.37
Debris in water	6 (30)	2 (2)	4 (4.0)	1	1
*Is your water stored before use?*					
Yes	183 (91.5)	97 (97)	86 (86.0)	0.0993	5.3
No	17 (8.5)	3 (3.0)	14 (14.0)	0.0447	0.28
*If yes, do you see sediments at the bottom?*					
Yes	127 (63.5)	50 (50)	77 (77.0)	<0.0001	0.298
No	53 (26.5)	47 (47.0)	6 (6.0)	<0.0001	13.9
Sometimes	3 (1.5)	0 (0)	3 (3.0)	0.081	0.139
*Does your water have taste?*					
Yes	42 (21.0)	7 (7.0)	35 (35.0)	<0.0001	0.14
No	158 (79)	93 (93.0)	65 (65.0)	<0.0001	8.86
Sometimes	5 (2.5)	0 (0)	5 (5.0)	0.0235	0.09
*Does your water smell?*					
Yes	160 (80)	97 (97.0)	63 (63.0)	<0.0001	19
No	40 (20.0)	3 (3.0)	37 (37.0)	<0.0001	0.053
*Does your water have colour?*					
Yes	65 (32.5)	65 (65)	0 (0)	<0.0001	0.003
No	135 (67.5)	96 (100)	39 (39.0)	<0.0001	0.003
*Do you drink from any other source?*					
Yes	100 (50)	37 (37.0)	63 (63.0)	<0.0001	0.22
No	100 (50)	63 (63.0)	27 (27.0)	0.0002	2.9
Don't know	1 (0.5)	1 (1.0)	0 (0)	0.3161	3.03

OR refers to odds ratio; *p* value refers to level of significance. The “far from the dam” communities were Agblekame North and Agblekame South while “near to the dam” communities were Gyama Nsuo-ano and Bongase nsuo-ano.

**Table 3:** Respondents' general knowledge of water usage patterns, water related tropical diseases, and behavioural change.

Variables	% total (200)	% far from the dam communities (100)	% near to the dam communities (100)	*p* value	Odd ratio
*How often do you come into contact with water?*					
Daily	23 (11.5)	18 (18.0)	5 (5.0)	0.004	0.24
Monthly	175 (87.5)	95 (95.0)	80 (80.0)	0.0013	4.8
None	2 (10)	0 (0)	2 (2.0)	0.1552	0.2
*Do you perceive any health problem from your water?*					
Yes	183 (91.5)	97 (97.0)	86 (86.0)	0.0993	5.3
No	17 (8.5)	3 (3.0)	14 (14.0)	0.0447	0.28
*Do you know anyone suffering from any of these diseases?*					
Malaria	16 (8.0)	12 (12.0)	4 (4.0)	0.04	3.27
Diarrhea	36 (18.0)	11 (11.0)	25 (25.0)	0.01	0.37
Onchocerciasis	10 (5.0)	8 (8.0)	2 (2.0)	0.05	4.3
Schistosomiasis	25 (12.5)	2 (2.0)	23 (23.0)	<0.0001	0.7
*What are possible symptoms observed?*					
Coughing	20.0 (10.0)	9 (9.0)	11 (11.0)	0.0637	0.37
Diarrhea	56 (28.0)	19 (19.0)	37 (37.0)	0.0046	0.399
Blood in urine	100 (55.0)	43 (43.0)	67 (67.0)	0.0006	0.8
Impaired vision	4 (2.0)	1 (1.0)	3 (3.0)	0.312	0.33
None	10 (5.0)	5 (5.0)	5 (5.0)	1	1
*How often do you wash your hands in a day?*					
2 times	42 (21)	7 (7.0)	35 (35.0)	<0.0001	0.14
3 times	158 (79)	65 (65.0)	93 (93.0)	<0.0001	8.86
More than 3 times	5 (2.5)	0 (0)	5 (5.0)	0.0235	0.09
*What do you wash your hand with*					
Soap and water	25 (12.5)	2 (2.0)	23 (23.0)	<0.0001	0.07
Only water	84 (42)	34 (34.0)	50 (50.0)	0.0219	0.52
*When is the washing done?*					
After eating	106 (53)	33 (33.0)	73 (73.0)	<0.0001	0.182
After visiting the toilet	93 (46.5)	66 (66.0)	27 (27.0)	<0.0001	5.25
After daily work	1 (0.5)	1 (1.0)	0 (0)	0.3161	3.03
*Why and how do you sometimes treat your drinking water?*					
When it smells bad	0	0 (0)	0 (0)	0 (0)	0
By filtration to make clean	0	0 (0)	0 (0)	0 (0)	0
By boiling to remove germs	109 (54.5)	21 (21.0)	88 (88.0)	<0.0001	0.03
By boiling reduce chances of disease	81 (40.5)	69 (69.0)	12 (12.0)	<0.0001	0.06
*How do you keep left over food?*					
Reheat	81 (40.5)	69 (69.0)	12 (12.0)	<0.0001	0.06
In the room	3 (1.5)	0 (0)	3 (3.0)	0.081	0.14
Covered	109 (54.5)	21 (21.0)	88 (88.0)	<0.0001	0.03
*Where do you store the food?*					
Covered container	160 (80)	97 (97.0)	63 (63.0)	<0.0001	19
Uncovered container	40 (20.0)	3 (3.0)	37 (37.0)	<0.0001	0.053
None	0	0 (0)	0 (0)	0 (0)	0
*Why should food be stored when not eaten?*					
Keeps flies off	52 (26.0)	21 (21.0)	31 (31)	0.106	0.59
Prevent diseases	16 (8.0)	5 (5.0)	11 (11.0)	0.118	0.43
Keep food clean	6 (3.0)	2 (2.0)	4 (4.0)	0.41	0.49
Keep warm	56 (28.0)	23 (23.0)	33 (33.0)	0.115	0.6
*How many time do you bath in a day?*					
One time	20 (10)	11 (11.0)	9 (9.0)	0.64	0.8
Two times	170 (0.85)	94 (94.0)	76 (76.0)	0.15	3.13
Never	3 (1.5)	3 (3.0)	0 (0)	0.081	0.14
*How do you dispose off your solid waste?*					
Burn	102 (51)	15 (15.0)	87 (87.0)	<0.0001	0.03
Community dump site	6 (6.0)	2 (2.0)	4 (4.0)	1	1
Left in the open	30 (15.0)	24 (24.0)	6 (6.0)	0.0004	4.94
*How do you dispose of your liquid waste?*					
Lead to drain	93 (46.5)	27 (27.0)	66 (66.0)	<0.0001	0.3
Thrown on the ground	53 (26.5)	47 (47.0)	6 (6.0)	<0.0001	13.9
Thrown into the river	3 (1.5)	0 (0)	3 (3.0)	0.081	0.14

OR refers to odds ratio; *p* value refers to level of significance. The “far from the dam” communities were Agblekame North and Agblekame South while “near to the dam” communities were Gyama Nsuo-ano and Bongase nsuo-ano.

## Data Availability

The authors declare that data for this work will be available upon reasonable request.
